# The N-terminus of Mcm10 is important for interaction with the 9-1-1 clamp and in resistance to DNA damage

**DOI:** 10.1093/nar/gku479

**Published:** 2014-06-27

**Authors:** Robert C. Alver, Tianji Zhang, Ajeetha Josephrajan, Brandy L. Fultz, Chance J. Hendrix, Sapna Das-Bradoo, Anja-Katrin Bielinsky

**Affiliations:** 1 Department of Biochemistry, Molecular Biology and Biophysics, University of Minnesota, Minneapolis, MN 55455, USA; 2 Department of Natural Sciences, Northeastern State University, 3100 East New Orleans Street, Broken Arrow, OK 74012, USA

## Abstract

Accurate replication of the genome requires the evolutionarily conserved minichromosome maintenance protein, Mcm10. Although the details of the precise role of Mcm10 in DNA replication are still debated, it interacts with the Mcm2-7 core helicase, the lagging strand polymerase, DNA polymerase-α and the replication clamp, proliferating cell nuclear antigen. Loss of these interactions caused by the depletion of Mcm10 leads to chromosome breakage and cell cycle checkpoint activation. However, whether Mcm10 has an active role in DNA damage prevention is unknown. Here, we present data that establish a novel role of the N-terminus of Mcm10 in resisting DNA damage. We show that Mcm10 interacts with the Mec3 subunit of the 9-1-1 clamp in response to replication stress evoked by UV irradiation or nucleotide shortage. We map the interaction domain with Mec3 within the N-terminal region of Mcm10 and demonstrate that its truncation causes UV light sensitivity. This sensitivity is not further enhanced by a deletion of *MEC3*, arguing that *MCM10* and *MEC3* operate in the same pathway. Since Rad53 phosphorylation in response to UV light appears to be normal in N-terminally truncated *mcm10* mutants, we propose that Mcm10 may have a role in replication fork restart or DNA repair.

## INTRODUCTION

Genome maintenance is one of the highest priorities for all eukaryotic organisms, a task confounded by the constant assault of intrinsic and environmental factors that damage an organism's DNA. To counteract these factors, cells employ a DNA damage response (DDR), a complex network of signaling proteins that can sense compromised DNA and activate appropriate cellular processes depending on the type and amount of the damage (1). As dysfunction in the DDR or the pathways it activates can result in genome instability (2), one of the distinguishing features of cancer, we are interested in understanding exactly which factors contribute to the maintenance of genome stability and how they carry out their functions.

Our laboratory and others have established minichromosome maintenance (Mcm) protein 10 as a strong protector of genome stability (3–8). Mcm10 is an essential, conserved replication factor (9,10) important in both the initiation and elongation steps of DNA replication. It accomplishes this through its interactions with several components of the core replication machinery, including the Mcm2-7 helicase (11), polymerase-α (Polα)/primase (3,12–14) and the replication clamp, proliferating cell nuclear antigen (PCNA) (15). Loss of Mcm10 protein expression results in induction of DNA breaks as well as checkpoint activation (3,16).

The Mcm10 protein has three major domains: an N-terminal domain containing a coiled-coil motif, an internal domain consisting of an oligonucleotide/oligosaccharide (OB) fold and a zinc finger, and a C-terminal domain that is unique to higher eukaryotes and which contains two zinc fingers (17,18). These domains are connected by regions with little sequence conservation or predicted secondary structure. Mcm10 forms oligomers through its N-terminus, although the functional relevance of this self-interaction is still unknown (17,19–21). The internal domain is well conserved across species, and Mcm10's interaction surfaces for Polα and PCNA have both been mapped to reside within the OB fold (15,22). Polα binds to an Hsp10-like domain on Mcm10 (22), whereas PCNA binds to Mcm10's PIP box, through which Mcm10 manifests its essential interaction with PCNA (15). In contrast to Mcm10's conserved core, its N- and C-terminal domains are less well understood.

In response to DNA damage or replication stress, cells employ a signaling cascade driven by two phosphoinositide (PI)-3-kinase-related kinases, Mec1 and Tel1 (23). While Tel1 activation is limited primarily to double-strand break signaling, activation of Mec1 occurs in response to DNA damage and replication stress. Multiple proteins are independently recruited to DNA and collaborate to activate Mec1, including Dpb11 and the heterotrimeric 9-1-1 checkpoint clamp, consisting of the *Saccharomyces cerevisiae* proteins Ddc1, Mec3 and Rad17 (24–27). The 9-1-1 clamp is required for signaling DNA damage and specifically binds to 5' recessed DNA (28,29). It shares significant structural homology with PCNA (30,31), although they have divergent sequences and functions. Because of this structural similarity and Mcm10's clear role in protecting genome stability, we investigated whether Mcm10 played any part in checkpoint activation via an interaction with 9-1-1. Here we show that Mcm10 interacts with the Mec3 subunit of the 9-1-1 clamp and we map the interaction sites to Mcm10's N-terminus and PIP box, and Mec3's interdomain loop (IDL). Elimination of the N-terminal Mec3-binding domain in Mcm10 results in a checkpoint activation defect when cells are exposed to hydroxyurea (HU), but not in the presence of DNA damage. Nonetheless, truncation of the N-terminal binding site results in sensitivity to DNA-damaging agents in a Mec3-dependent and -independent manner, contingent on the type of genotoxic agent. These data suggest that Mcm10 may act as a protein scaffold that connects the DNA replication, checkpoint activation and DNA repair machineries to protect genome stability.

## MATERIALS AND METHODS

### Strains and plasmids

All strains used in this study are derived from W303-1a (Table 1). Plasmids were generated using standard molecular cloning techniques and point mutations were created using a QuikChange Lightning Site Directed Mutagenesis Kit (Agilent Technologies). Plasmids for the yeast two-hybrid analysis were constructed in the pBTM116 and pACT2 vectors (15). Integration plasmids used to C-terminally tag Mcm10-9Myc and Mec3-3HA were generated using standard cloning techniques. The coding sequence for Mcm10, as well as the N-terminal truncation mutants, was cloned into the pRS316 plasmid in addition to 372 bp of upstream promoter sequence. All gene deletions were made using polymerase chain reaction-based gene disruption methods (32).

**Table 1.** Yeast strains utilized in this study

Strain name	Relevant genotype	Source
All strains derived from AByb021 (W303-1a)	*MATa ura3-1 ade 2-1 his3-11,-15 leu2-3,-112 can1-100 trp1-1 BAR1::LEU2 rad5-535*	(15)
AByb509	*MEC3::MEC3-3HA*	This study
AByb1632	*MCM10::MCM10-9MYC*	
	*MEC3::MEC3-3HA*	This study
AByb1487	*MCM10::HIS3*, p*RS316-MCM10-2HA*	This study
AByb1489	*MCM10::HIS3*, p*RS316-mcm10Δ100-2HA*	This study
AByb1491	*MCM10::HIS3*, p*RS316-mcm10Δ150-2HA*	This study
AByb1517	*MCM10::HIS3*, p*RS316-mcm10Δ150-2HA, MEC3::TRP1*	This study
AByb1576	*MCM10::HIS3*, p*RS316-MCM10-2HA, MEC3::TRP1*	This study
AByb1594	*MCM10::HIS3*, p*RS316-mcm10Δ100-2HA, MEC3::TRP1*	This study
AByb925	*DDC1: DDC1-3HA*	
	*MCM10::MCM10-9MYC*	This study
AByb1961	*DDC1: DDC1-3HA*	This study
AByb1964	*MCM10::HIS3*, p*RS316-MCM10-2HA*	
	*MEC3::MEC3-3HA*	This study
AByb1967	*MCM10::HIS3*, p*RS316-MCM10-2HA*	
	*MEC3::mec3-LLI-3HA*	This study
AByb1968	*MCM10::HIS3*, p*RS316-mcm10Δ100-2HA*	
	*MEC3::mec3-LLI-3HA*	This study
AByb1969	*MCM10::HIS3*, p*RS316-mcm10Δ150-2HA MEC3::mec3-LLI-3HA*	This study
ABy2080	p*BTM116-LEXA-MCM10*, p*ACT2-MEC3*	This study
ABy2081	p*BTM116-LEXA-mcm10Δ100*, p*ACT2-MEC3*	This study
ABy2081	p*BTM116-LEXA-mcm10Δ150*, p*ACT2-MEC3*	This study

### Protein preparation and immunoblotting

Total protein extracts were obtained from yeast cultures by trichloroacetic acid (TCA) preparation as described and were separated by sodium dodecylsulphate-polyacrylamide gel electrophoresis (SDS-PAGE) and subsequently transferred onto nitrocellulose membrane (33). Hemagglutinin (HA)-tagged Mec3 was visualized using a horseradish peroxidase (HRP)-conjugated anti-HA antibody (Roche, 3F10). Expression of genes cloned into the pBTM116 vector were detected with an anti-LexA antibody (Abcam, ab14553), and those cloned into the pACT2 vector were detected using an anti-HA antibody (Covance, 16B12). Myc-tagged Mcm10 was detected using an anti-Myc antibody (Thermo Scientific, 9E11), and endogenous Rad53 was detected using an anti-Rad53 antibody (gift from Dr J.F.X. Diffley).

### DNA damage treatment and replication stress induction

A 500-ml culture was grown until mid-log phase. It was then subdivided into 100-ml aliquots and treated with 200-mM HU, 100 μg/ml phleomycin (PLE) or 100-μM camptothecin (CPT) for 1 h. For ultraviolet (UV) treatment, 10-ml aliquots were spread on a 150 × 15 mm petri dish, irradiated (100 J/m^2^), pooled and allowed to recover for 1 h. Protein was precipitated by TCA or whole cell extracts (WCEs) were prepared as described below.

### Whole cell extraction for co-immunoprecipitation

WCEs were made by resuspending cell pellets in lysis buffer [50-mM HEPES/KOH pH 7.5, 140-mM NaCl, 1-mM ethylenediaminetetraaceticacid, 1% Triton X-100, 0.1% sodium deoxycholate, 1-μM phenylmethylsulfonyl fluoride (PMSF), 1-μM benzamidine, 2-μM leupeptin, 1-μM pepstatin and 5-mM *N*-ethylmaleimide (NEM)], adding an equal volume of acid-washed glass beads and vortexing for 5 min at 4°C. Extracts were clarified by centrifugation for 1 min at 4°C at 1500 x g and total protein content was estimated using a Bradford assay. Equal amounts of protein were treated with 150-kU DNase I (Sigma) in the presence of 10-mM MgCl_2_ for 30 min on ice, and were used for subsequent co-immunoprecipitation (Co-IP) experiments.

### Co-IP experiments

DNaseI-treated WCEs were diluted with 100-μl IP buffer (20-mM Tris pH 7.4, 150-mM NaCl, 0.1% Igepal CA-630) and incubated with 50 μl of a 1:1 suspension of anti-c-Myc (Sigma, A7470), 10 μl of a 1:1 suspension of anti-LexA (Santa Cruz Bio, sc-1762) or 20 μl of a 1:1 suspension of IgG agarose beads (Sigma, A0919) for 1 h at 4°C. The beads were washed once briefly, and twice for 5 min with rotation at room temperature with IP buffer that contained 200-mM NaCl and had been supplemented with 1-μM PMSF, 1-μM benzamidine, 2-μM leupeptin, 1-μM pepstatin and 5-mM NEM. Proteins were eluted using SDS loading buffer, separated by SDS-PAGE and visualized by immunoblot.

### β-Galactosidase activity

Yeast two-hybrid assays were performed as described (15). Briefly, 10-ml overnight cultures of cells harboring the indicated bait and prey plasmids were collected, washed first with 25 ml of ice-cold buffer P [50-mM sodium phosphate (pH 7.7), 300-mM sodium acetate, 10% glycerol, 1-mM 2-mercaptoethanol, 500-nM dithiothreitol, 1 μg/ml pepstatin, 1-mM PMSF, 1-mM benzamidine, 0.5 μg/ml leupeptin, 5-mM NEM] and then a second time with 1 ml buffer P. Cells were resuspended in 200 μl buffer P and subsequently lysed by vortexing with acid-washed glass beads. Extracts were clarified by centrifugation and the supernatant was tested for β-galactosidase activity using a β-galactosidase activity assay kit (Invitrogen). β-Galactosidase activity and total protein, measured by a Bradford assay, were determined in triplicate from at least three individual clones for each two-hybrid strain. The interaction between Pol32 and PCNA served as a positive control.

### Serial dilution assays

The indicated strains were grown for 2–3 days and 2 × 10^7^ cells were resusupended in 300-μl sterile water and pipetted into the first column of a 96-well plate. Serial, 10-fold dilutions were made in each successive column of the plate. Cells were transferred from the plate to the indicated growth medium using a 48-spoke inoculating manifold, and the plates were grown for 3–5 days at 30°C.

### Colony survival assay

The assay for camptothecin (CPT) sensitivity was performed essentially as described (34). Briefly, 10-ml logarithmic cultures were treated with the indicated concentrations of CPT for 6 h. Aliquots of each strain and condition were plated in triplicate onto synthetic complete medium lacking uracilif (SC-URA) and grown for 2–4 days. Colonies were counted and survival was normalized to an untreated control. Average percentage survival for each strain and condition are reported.

## RESULTS

### Mcm10 interacts with the 9-1-1 clamp through the Mec3 subunit

**Figure 1.**
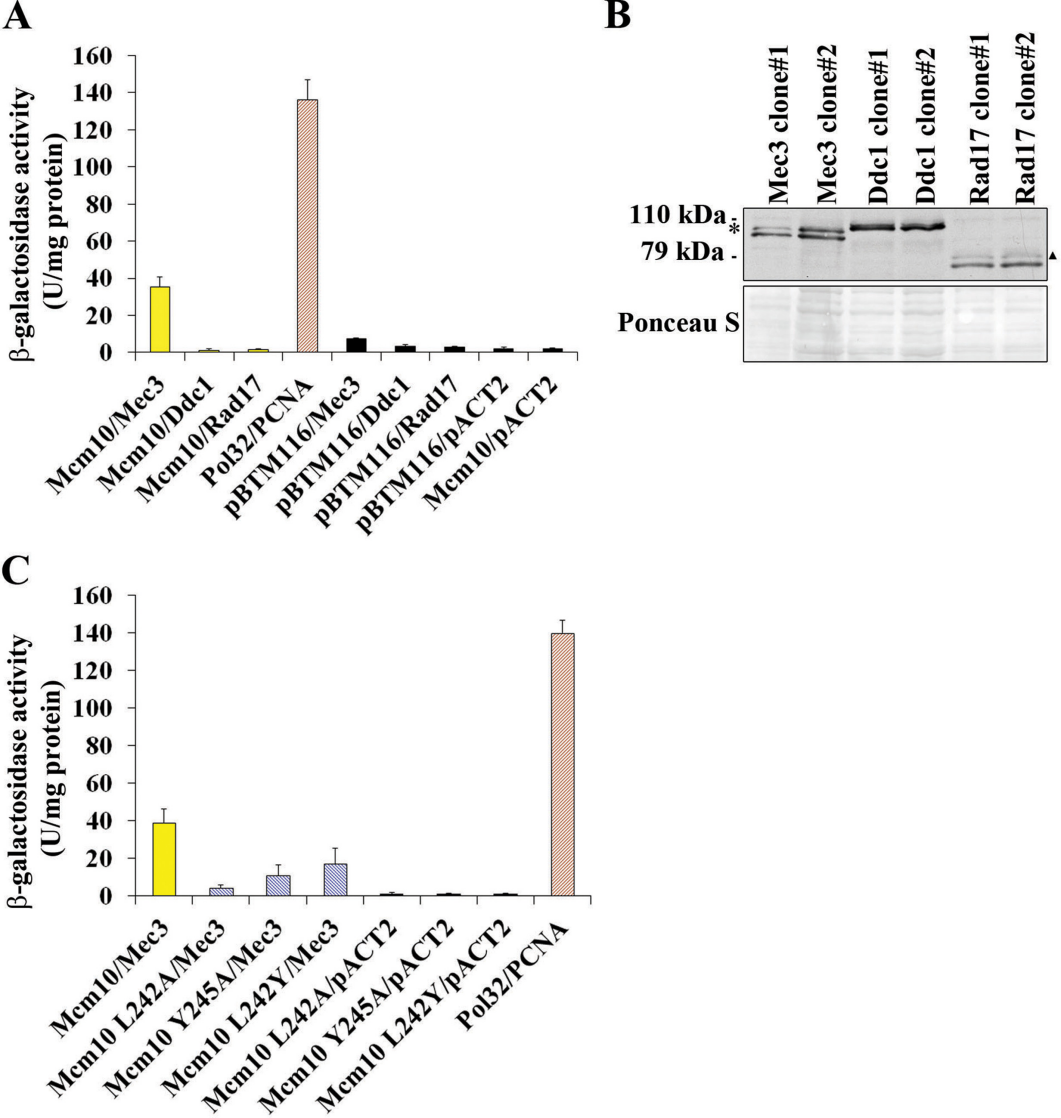
Mcm10 interacts with the Mec3 subunit of the 9-1-1 clamp partially through its PIP box. (**A**) β-Galactosidase activity was measured in cell extracts obtained from yeast two-hybrid strains expressing Mec3, Rad17 and Ddc1 fusion proteins that co-expressed Mcm10. Pol32 and PCNA served as a positive control, and extracts expressing the pACT2 and pBTM116 empty vectors served as a negative control. Each combination was tested in triplicate with three individual transformants. Error bars indicate standard deviations. (**B**) Immunoblot using an anti-HA antibody (Covance, 16B12) indicates proper expression of the Mec3, Ddc1 and Rad17 fusion proteins. The asterisk and triangle indicate bands of uncertain origin. Ponceau S staining served as a loading control. (**C**) β-Galactosidase activity was measured in cell extracts obtained from yeast two-hybrid strains expressing Mcm10 L242A, Mcm10 L242Y and Mcm10 Y245A fusion proteins that co-expressed Mec3. Pol32 and PCNA served as a positive control, and extracts expressing the pACT2 empty vector served as a negative control. Each combination was tested in triplicate with three individual transformants. Error bars indicate standard deviations.

We previously described an interaction between Mcm10 and PCNA (15). To investigate whether Mcm10 also interacts with any of the 9-1-1 clamp subunits, we performed yeast two-hybrid assays. Each 9-1-1 subunit, Ddc1, Mec3 and Rad17, was tested against Mcm10 or an empty vector control. The relative strength of each interaction was quantified by a β-galactosidase activity assay. We were able to detect a moderate level of binding between the Mec3 subunit of the 9-1-1 clamp and Mcm10, but not between Mcm10 and Ddc1 or Rad17 (Figure 1A). The expression of all constructs was confirmed by immunoblotting (Figure 1B and Supplementary Figure S1) to ensure that the lack of an interaction was not due to a lack of protein expression. The relative strength of the interaction between Mcm10 and Mec3 was similar to the level observed between diubiquitinated Mcm10 and PCNA (15) and was significantly higher than the negative controls. To determine if Mcm10 and Mec3 bind at the molecular level in a manner similar to Mcm10 and PCNA, we employed Mcm10 PIP box mutants, L242A, Y245A and L242Y, which each disrupts the interaction between Mcm10 and PCNA. As documented in an earlier report, all of these mutants reduced the interaction with PCNA to background levels (15). When we tested these mutants for binding to Mec3, the interaction was significantly reduced (Figure 1C). The L242Y mutant displayed a slightly stronger residual interaction than the other two mutants (Figure 1C), similar to Mcm10 PIP box mutants and PCNA (15). Together, these results argue that the interaction between Mcm10 and Mec3 is partially mediated through Mcm10's PIP box.

### The N-terminus of Mcm10 facilitates binding to Mec3

**Figure 2.**
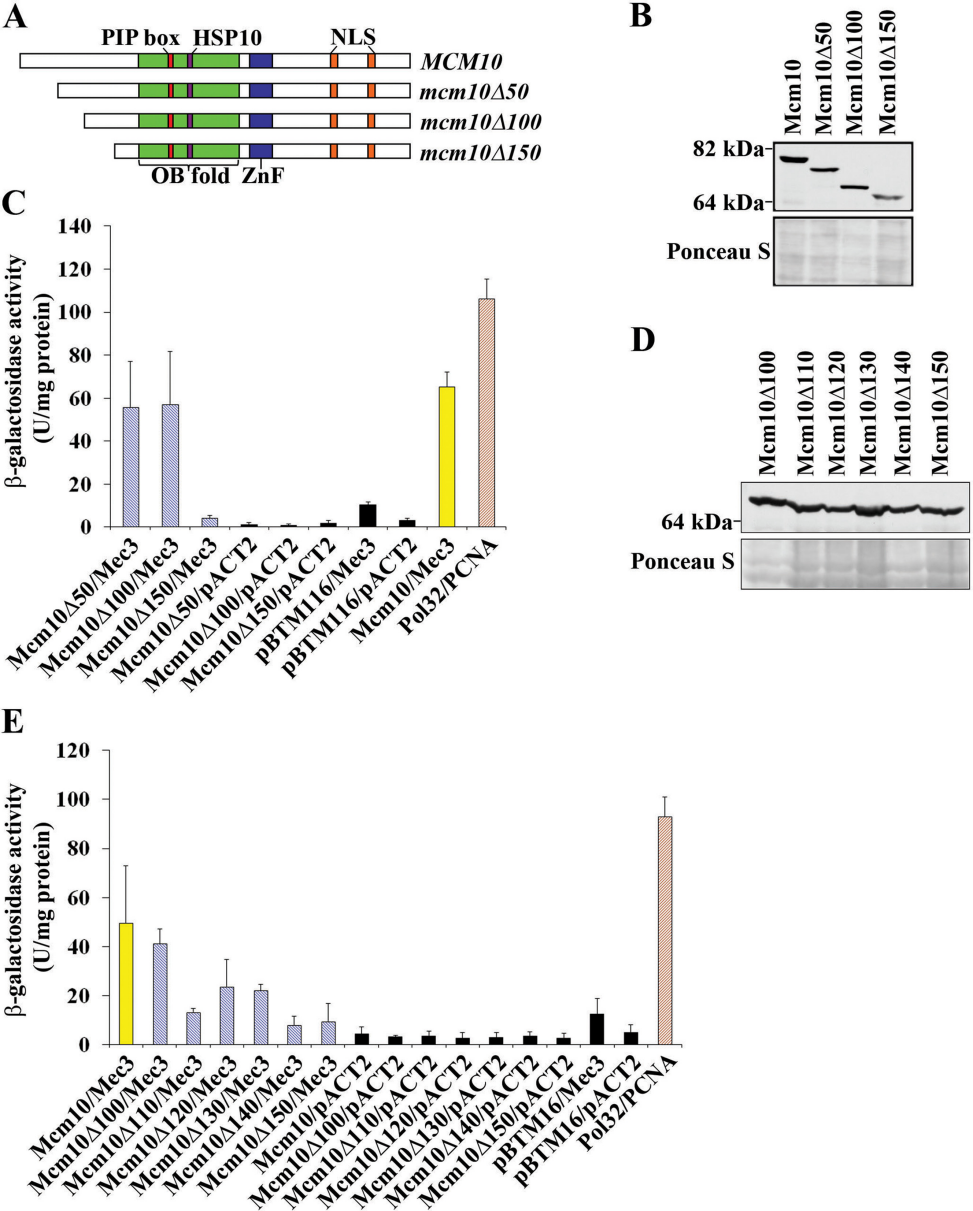
The Mcm10:Mec3 interaction is disrupted by a 150 amino acid N-terminal truncation. (**A**) Schematic representation of known Mcm10 domains and motifs and the regions that were truncated. (**B**) Immunoblot using an anti-LexA antibody (Abcam, ab14553) indicates proper expression of Mcm10 wild-type, Δ50, Δ100 and Δ150 truncation proteins. Ponceau S staining served as a loading control. (**C**) β-Galactosidase activity was measured in cell extracts obtained from yeast two-hybrid strains expressing Mcm10, Mcm10Δ50, Mcm10Δ100 and Mcm10Δ150 fusion proteins that co-expressed Mec3. Pol32 and PCNA served as a positive control, and extracts expressing the pACT2 and pBTM116 empty vectors served as negative controls. Each combination was tested in triplicate with three individual transformants. Error bars indicate standard deviations. (**D**) Immunoblot using an anti-LexA antibody (Abcam ab14553) indicates proper expression of Mcm10Δ100, Mcm10Δ110, Mcm10Δ120, Mcm10Δ130, Mcm10Δ140 and Mcm10Δ150 truncations. Ponceau S staining served as a loading control. (**E**) β-Galactosidase activity was measured in cell extracts obtained from yeast two-hybrid strains expressing Mcm10, Mcm10Δ100, Mcm10Δ110, Mcm10Δ120, Mcm10Δ130, Mcm10Δ140 and Mcm10Δ150 fusion proteins that co-expressed Mec3. Pol32 and PCNA served as a positive control, and extracts expressing the pACT2 and pBTM116 empty vectors served as negative controls. Each combination was tested in triplicate with three individual transformants. Error bars indicate standard deviations.

**Figure 3.**
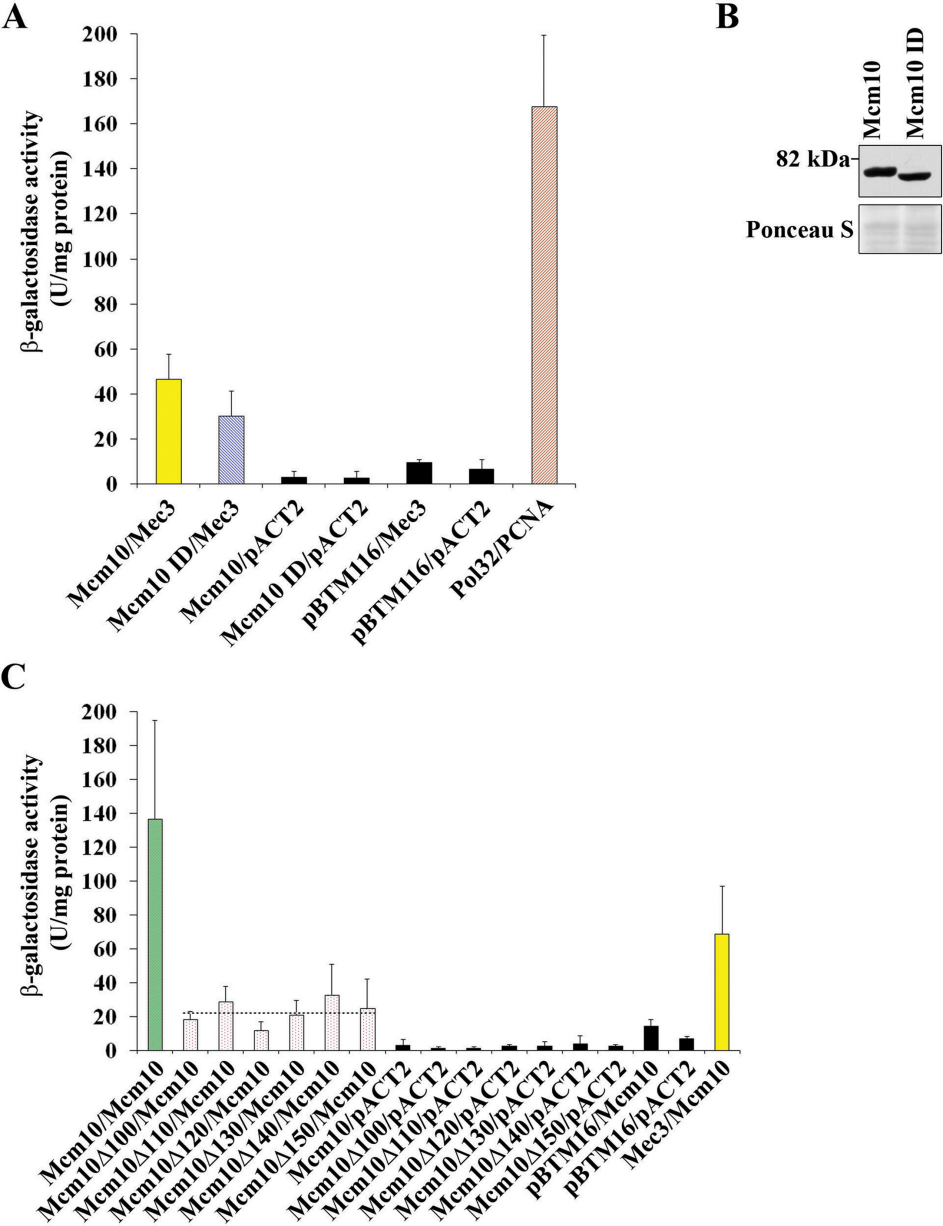
An ID of amino acids 100–150 does not eliminate binding to Mec3, but Mcm10 self-interaction is eliminated by truncation of the N-terminal 100 amino acids. (**A**) β-Galactosidase activity was measured in cell extracts obtained from yeast two-hybrid strains expressing Mcm10, and the Mcm10 ID fusion proteins that co-expressed Mec3. Pol32 and PCNA served as a positive control, and extracts expressing the pACT2 and pBTM116 empty vectors served as negative controls. Each combination was tested in triplicate with three individual transformants. Error bars indicate standard deviations. (**B**) Immunoblot using an anti-HA antibody (Covance, 16B12) indicated proper expression of the Mec3 ID. Ponceau S staining served as a loading control. (**C**) β-Galactosidase activity was measured in cell extracts obtained from yeast two-hybrid strains expressing Mcm10, Mcm10Δ100, Mcm10Δ110, Mcm10Δ120, Mcm10Δ130, Mcm10Δ140 and Mcm10Δ150 fusion proteins that co-expressed Mcm10. Pol32 and PCNA, and Mcm10 tested with Mec3 served as positive controls, and extracts expressing the pACT2 and pBTM116 empty vectors served as negative controls. Each combination was tested in triplicate with three individual transformants. Error bars indicate standard deviations.

We sought to further characterize the binding between Mcm10 and Mec3 in the hopes of identifying a separation-of-function mutant of Mcm10 that would retain its ability to interact with PCNA, but would be deficient in Mec3 binding. Toward this end, we constructed N-terminal truncation mutants of Mcm10 that left the PIP box intact, but deleted the first 50, 100 or 150 amino acids (Figure 2A). This region of Mcm10 lies outside the conserved OB-fold/Zn-finger domain and is not predicted to have any significant secondary structure (17), except for a coiled-coil region at the very N-terminus (21). All fusion proteins were expressed (Figure 2B and Supplementary Figure S1) and we then assessed binding to Mec3 via two-hybrid analyses (Figure 2C). Loss of the N-terminal 50 or 100 residues was inconsequential for Mec3 binding, but deletion of the next 50 amino acids resulted in a near complete loss of interaction (Figure 2A–C). A second series of constructs was then designed to further narrow down the Mec3-interaction domain between amino acids 100 and 150 of Mcm10 (Mcm10Δ110, McmΔ120, McmΔ130 and McmΔ140) and each construct's expression was confirmed (Figure 2D). The individually truncated proteins showed intermediate levels of interaction and did not clearly implicate any specific subregion in Mec3 binding (Figure 2E). Because the Mcm10Δ150 protein eliminated Mec3 binding in two-hybrid assays, we attempted to construct a mutant that left at least a portion of the N-terminus intact, as the N-terminal domain is important for Mcm10 oligomerization (17,21). Unfortunately, an internal deletion (ID) of residues 100–150 of Mcm10 failed to fully eliminate the interaction with Mec3 (Mcm10 ID in Figure 3A and B). Resigned to using the Mcm10Δ150 mutant in further experiments, we sought to determine this mutant's ability to bind to wild-type Mcm10. Mcm10 binding to the Mcm10Δ150 mutant was reduced by ∼85%, and is similarly reduced in the Mcm10Δ100 and all Mcm10 N-terminal truncation mutants spanning residues 100–150 (Figure 3C). Importantly, the Mcm10Δ100 and Mcm10Δ150 truncation proteins fully retained the capacity to bind PCNA (Supplementary Figure S2), arguing that the inability of Mcm10Δ150 to recognize Mec3 was not due to a distortion of the PIP box. Taken together, we conclude that the N-terminus of Mcm10 harbors a second Mec3 binding site that resides within amino acids 100–150. However, this binding site does not appear to be unique, but can be substituted by redundant sites in the N-terminus. Thus, the most reliable way for us to disrupt the interaction between Mcm10 and Mec3 was by deleting all redundant sites within the first 150 amino acids. Since this truncation also eliminated Mcm10:Mcm10 self-interaction, we included an *mcm10*Δ*100* mutant into our subsequent genetic analyses to distinguish between phenotypes caused by the loss of self-interaction or the combined loss of self-interaction and Mec3 binding.

### The interdomain loop of Mec3 is critical for its interaction with Mcm10

**Figure 4.**
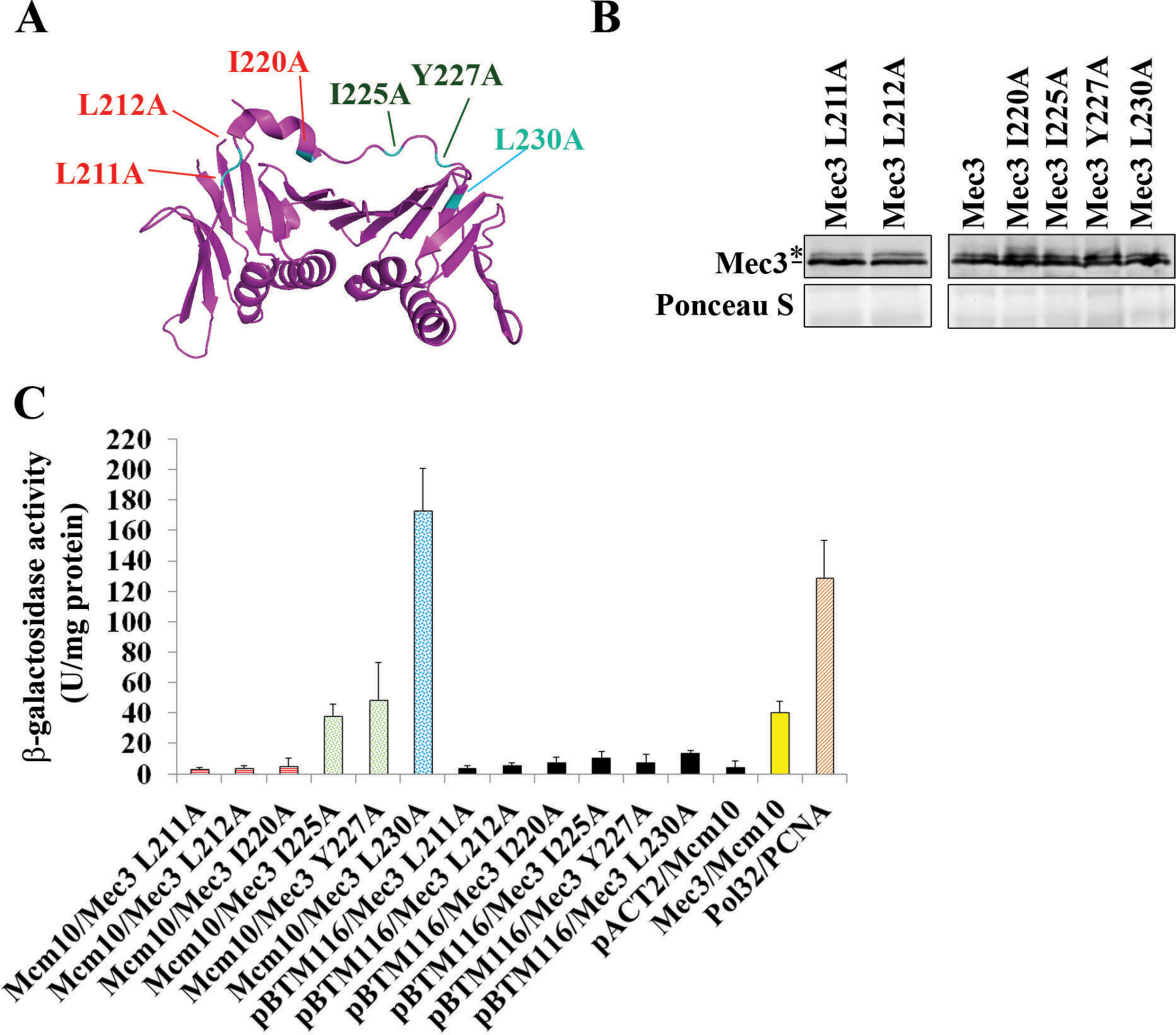
The IDL of Mec3 mediates its interaction with Mcm10. (**A**) Visualization of the Mec3-homolog, hsHus1 subunit of 9-1-1 and estimation of the location of Mec3 L211A, Mec3 L212A, Mec3 I220A, Mec3 I225A, Mec3 Y227A and Mec3 L230A point mutants constructed for the yeast two-hybrid assay. PDB file 3G65 (30) Graphics obtained using PyMol, The PyMOL Molecular Graphics System, Version 1.2r3pre, Schrödinger, LLC. (**B**) Immunoblot using an anti-HA antibody (Covance, 16B12) indicates proper expression of the indicated Mec3 IDL mutants. The asterisk indicates a band of uncertain origin, which may be a modified form of Mec3. Ponceau S staining served as a loading control. (**C**) β-Galactosidase activity was measured in cell extracts obtained from yeast two-hybrid strains expressing Mec3, Mec3 L211A, Mec3 L212A, Mec3 I220A, Mec3 I225A, Mec3 Y227A and Mec3 L230A fusion proteins that co-expressed Mcm10. Pol32 and PCNA served as positive control, and extracts expressing the pACT2 and pBTM116 empty vectors served as negative controls. Each combination was tested in triplicate with three individual transformants. Error bars indicate standard deviations.

To identify possible Mcm10 interaction sites in Mec3, candidate residues within the predicted Mec3 IDL were selected for mutation based on sequence alignment of Mec3 with homologous proteins, as well as PCNA (35). K210, L211, L212, I220, I225 and Y227 exhibited strong sequence conservation (Supplementary Figure S3) and were selected for mutation to alanine (Figure 4A). L230 was included to represent a residue that was not conserved. Mec3-K210A was unstable (data not shown), but the remainder of the IDL mutants was tested for binding to Mcm10 by two-hybrid assays, as they could be stably expressed (Figure 4B and Supplementary Figure S1). Three point mutations within the Mec3 IDL, L211A, L212A and I220A, significantly reduced Mec3's binding to Mcm10 (Figure 4C). Mutation of two nearby residues, I225A and Y227A, had little or no effect on binding of Mec3 to Mcm10, and much to our surprise, the mutation of a residue near the end of the IDL, L230A, resulted in a greatly increased ability of Mec3 to bind to Mcm10. We concluded from these data that residues within the IDL of Mec3 facilitate binding to Mcm10.

### The Mcm10:Mec3 interaction is stimulated by replication stress and DNA damage

**Figure 5.**
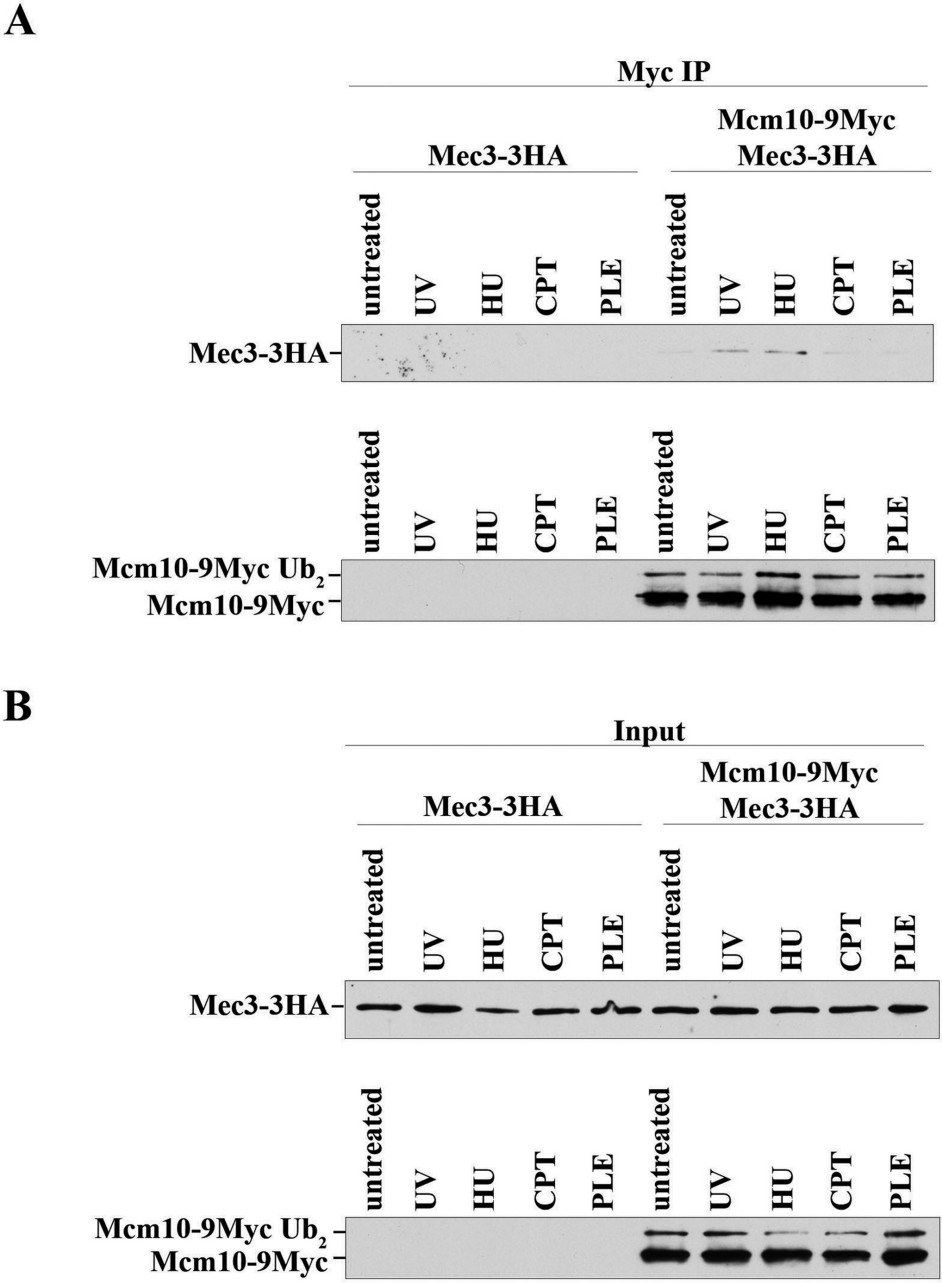
Mcm10 interacts with Mec3 after exposure of yeast cells to DNA-damaging agents or replication stress. AByb509 (*MEC3-3HA*) cells or AByb1632 (*MEC3-3HA*, *MCM10-9MYC*) cells were grown to log phase and treated with 100-J/m^2^ UV, 200-mM HU, 50-mg/ml PLE or 100-mM CPT as indicated for 1 h. Cells were harvested, WCEs were prepared and subjected to immunoprecipitation and proteins were detected by immunoblot using an anti-HA (Roche, 3F10) or anti-Myc antibody (Thermo Scientific, 9E11). IP lanes are shown in (**A**) and input lanes are shown in (**B**); the input lanes represent 1/20 of the IP lanes.

To substantiate the above-described two-hybrid interaction between Mec3 and Mcm10, we wished to determine if the two proteins would be detectable in a common complex by Co-IP. To this end, endogenous Mcm10 was tagged at the C-terminus with nine copies of the Myc epitope and endogenous Mec3 was tagged at its C-terminus with three copies of the HA epitope. Interaction between Mcm10 and Mec3 was not detectable in DNase I-treated WCEs from logarithmically growing cultures. However, when the cultures were exposed to the replication inhibitor HU or irradiated with UV light, we detected a weak but reproducible interaction (Figure 5A). In contrast, when cultures were treated with the topoisomerase I (TopoI) inhibitor, CPT or PLE, which causes single- and double-stranded DNA breaks (DSBs), we did not pull down any substantial amounts of Mec3. This was not due to inefficient immunoprecipitation, as all samples showed comparable amounts of both the unmodified and ubiquitinated form of Mcm10 (Figure 5A). To further substantiate these findings, we performed similar pull down experiments and analyzed another 9-1-1 component, Ddc1. These yielded almost identical results (Supplementary Figure S4). Together, these data indicate that the Mcm10:9-1-1 interaction is triggered only in the presence of UV light-induced DNA damage or depletion of dNTP pools. To confirm that binding to Mec3 depended on the N-terminal 150 amino acids of Mcm10, we attempted to utilize a plasmid-borne *mcm10Δ150-9MYC* transgene under the control of the endogenous *MCM10* promoter, but this construct was unable to complement a temperature-sensitive *mcm10-1* allele (data not shown). We obtained similar results when we expressed LexA-tagged Mcm10Δ150 from the endogenous *MCM10* promoter (Supplementary Figure S5). However, when overexpressed from the *ADH1* promoter LexA-tagged Mcm10Δ150 restored viability of *mcm10-1* mutants at non-permissive conditions and was, thus, functional (Supplementary Figure S5). For Co-IP, we overexpressed LexA-tagged Mcm10, Mcm10Δ100 or Mcm10Δ150 in combination with HA-tagged Mec3 and pulled down Mcm10 with an anti-LexA antibody. Cells were left untreated or exposed to UV light. We detected a basal level of interaction for full-length Mcm10 and Mcm10Δ100, likely as a result of overexpression. The binding to Mec3 was enhanced upon UV irradiation (Supplementary Figure S6). In contrast, Mcm10Δ150 pulled down very little Mec3 (Supplementary Figure S6). These results argue that residues 100–150 of Mcm10 are required for stable interaction with the checkpoint clamp.

### Checkpoint signaling in N-terminal *mcm10* deletion mutants is unaffected by DNA damage, but diminished when cells undergo deoxyribose nucleotide depletion

**Figure 6.**
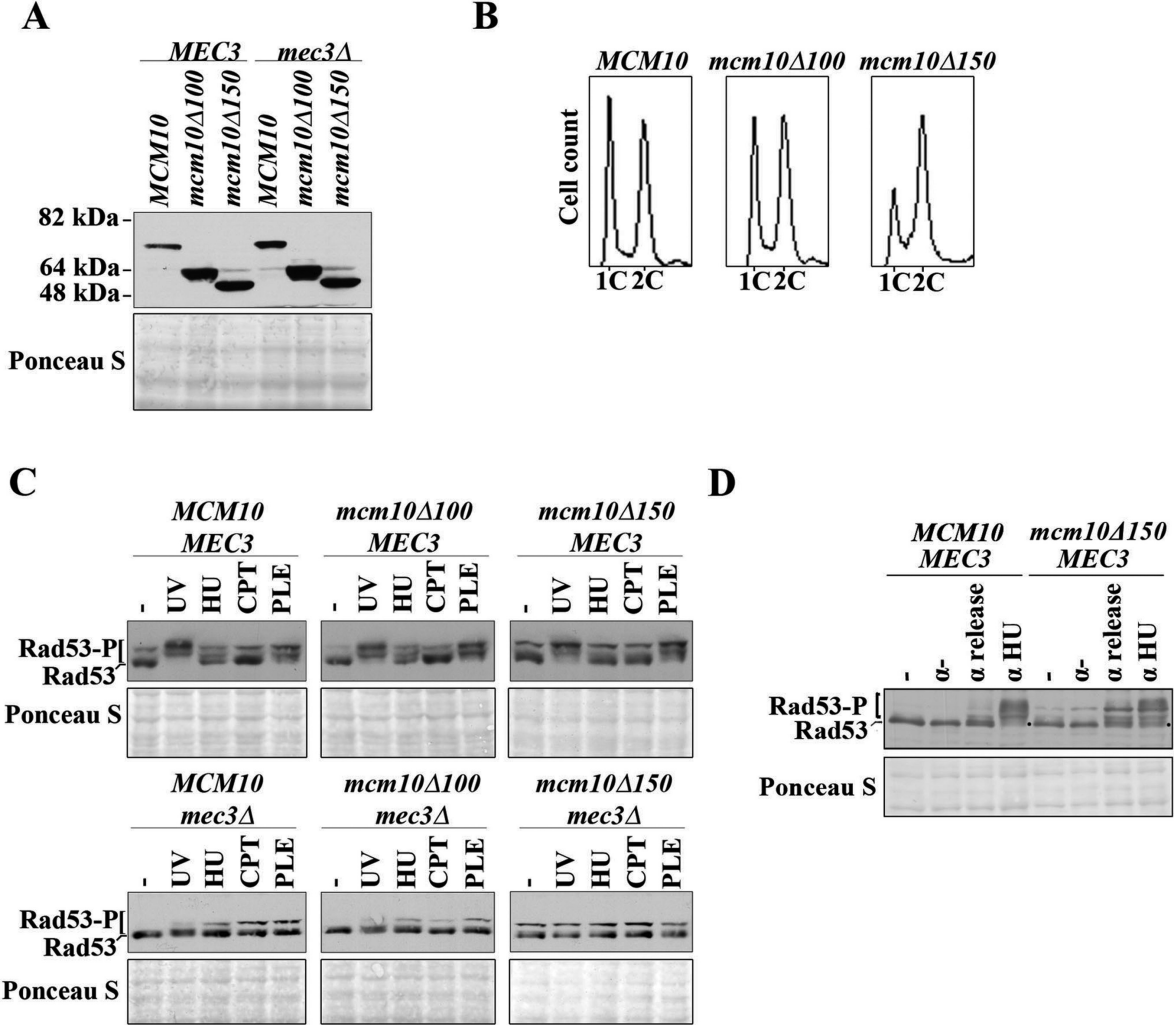
The *mcm10*Δ*150* strain is unable to mount a checkpoint response when undergoing replication stress, but has robust checkpoint activation in response to DNA-damaging agents. (**A**) Immunoblot using an anti-HA antibody (Roche, 3F10) indicates proper expression of the indicated Mcm10 truncation mutants. Ponceau S staining served as a loading control. (**B**) Fluorescence-activated cell sorting of asynchronous cultures of AByb1487 (*MCM10*), AByb1489 (*mcm10*Δ*100*) and AByb1491 (*mcm10Δ150*). (**C**) AByb1487 (*MCM10*), AByb1489 (*mcm10Δ100*), AByb1491 (*mcm10Δ150*), AByb1576 (*MCM10, mec3Δ*), AByb1594 (*mcm10Δ100, mec3Δ*) and AByb1517 (*mcm10Δ150, mec3Δ*) cells were either left untreated or treated with DNA-damaging agents and replication stress as described in ‘Materials and Methods', and protein was prepared by TCA extraction. Immunoblotting was used to detect Rad53 and its phosphorylated forms by an anti-Rad53 antibody. Ponceau S staining served as a loading control. (**D**) AByb1487 (*MCM10*) and AByb1491 (*mcm10Δ150*) cells were left untreated or synchronized with α-factor and released into either non-supplemented medium or medium containing 200-mM HU for 1 h. Cells were harvested, protein was prepared by TCA extraction and immunoblotting was used to detect Rad53 and its phosphorylated forms by an anti-Rad53 antibody. The two black dots indicate unmodified Rad53 in HU-treated cells. Ponceau S staining served as a loading control.

Because the 9-1-1 complex is important for the activation of cell cycle checkpoints (27), we assessed whether the elimination of the Mec3 binding region in the N-terminus of Mcm10 caused a deficiency in Rad53 phosphorylation in response to replication stress or DNA damage. To this end, we generated cells that expressed wild-type Mcm10, Mcm10Δ100 or Mcm10Δ150 under the control of the endogenous *MCM10* promoter from a transgene. The endogenous copy of *MCM10* was subsequently knocked out by gene replacement. All three genes showed robust expression, although the two truncation mutants displayed slightly higher steady-state levels than wild type (Figure 6A). The cell cycle profiles of logarithmic *MCM10* and *mcm10Δ100* cultures looked almost identical, whereas *mcm10Δ150* mutants showed some accumulation in S and G2 phase (Figure 6B). This slight cell cycle delay went hand-in-hand with a modest, but detectable activation of Rad53, which was independent of the 9-1-1 complex (Figure 6C, compare untreated lanes in the top and bottom panels). In addition, all strains were treated with UV light, HU, CPT or PLE as indicated in Figure 6C. Cells expressing wild-type Mcm10 or Mcm10Δ100 displayed normal, Mec3-dependent checkpoint activation in response to UV light, HU or PLE treatment (Figure 6C). As expected (36,37), exposure to CPT did not induce any significant Rad53 phosphorylation (Figure 6C). As alluded to above, in *mcm10Δ150* mutants, we observed low levels of checkpoint activation in asynchronous cultures as well as in cells that had been arrested in G1 phase with α-factor (Figure 6C and D). Activation of Rad53 was normal in response to UV light and PLE, but we noticed that the status of Rad53 phosphorylation in HU-treated cells looked identical to that of untreated cells (Figure 6C). To verify this reduction in checkpoint activation in response to HU treatment in the *mcm10Δ150* strain, we arrested cells in α-factor and released them into HU-containing or non-supplemented medium. In cells harboring the Mcm10Δ150 truncation, Rad53 phosphorylation was only slightly elevated following exposure to HU and a significant amount of Rad53 remained unmodified compared to wild-type cells (Figure 6D, compare signals marked by the black dots). Together, these data suggest that the N-terminal region of Mcm10 to which Mec3 binds in two-hybrid assays is not critical for checkpoint activation in response to UV light- or PLE-induced DNA damage, but it is required for full activation of Rad53 in the presence of HU.

### The N-terminus of Mcm10 is important for resistance to DNA damage

**Figure 7.**
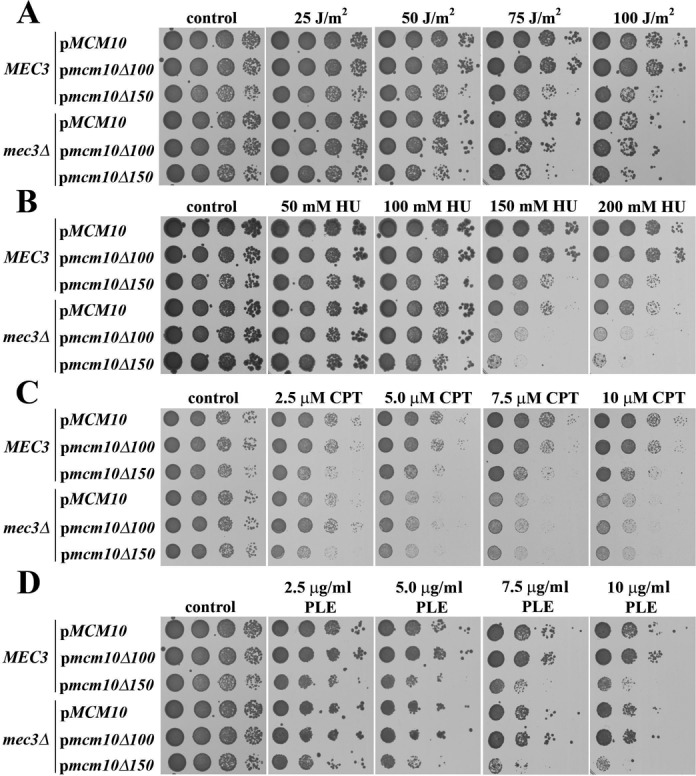
The N-terminus of Mcm10 is required for resistance to UV light, HU, CPT and PLE and acts in conjunction with Mec3, depending on the type of DNA damage. (**A**–**D**) Serial, 10-fold dilutions of AByb1487 (*MCM10*), AByb1489 (*mcm10Δ100*), AByb1491 (*mcm10Δ150*), AByb1576 (*MCM10, mec3Δ*), AByb1594 (*mcm10Δ100, mec3Δ*) and AByb1517 (*mcm10Δ150, mec3Δ*) were spotted on plates with the indicated concentrations of chemicals, or were treated with UV, and incubated at 30°C and plates were scanned after 3–5 days.

To extend on these results, we performed a serial dilution assay to assess growth on solid medium after UV irradiation or in the presence of HU, CPT or PLE. We compared strains harboring wild-type *MCM10*, *mcm10Δ100* or *mcm10Δ150* in a Mec3-proficient or deficient background. In the presence of Mec3, the *mcm10Δ150* allele caused a very slight growth delay (control panels in Figure 7A–D), consistent with our observation that Rad53 displayed a constitutive low-level activation in these cells (Figure 6C and D). To our surprise, *mcm10Δ150* mutants responded to each of the genotoxic treatments in a very different manner, whereas the *mcm10Δ100* strain mimicked cells expressing wild-type *MCM10* (Figure 7A–D). Sensitivity to UV light was significantly increased in cells harboring the *mcm10Δ150* mutation, but it was not exacerbated by a deletion of *MEC3*. The UV light-inflicted growth defect was similar to that observed in any of the *mec3Δ* cells (Figure 7A). To confirm this independently, we generated *mec3-LLI* mutants in which three residues of the Mec3 IDL, L211, L212 and I220, were substituted by alanines. These mutations had exhibited reduced binding to Mcm10 in two-hybrid assays (Figure 4C). Combinations of the *mec3-LLI* allele with N-terminal truncations of Mcm10 exhibited no further increase in UV sensitivity, similar to the *mec3Δ* strain (Supplementary Figure S7). Based on these findings, we concluded that the N-terminus of Mcm10 helps resist UV light-induced DNA damage and participates in the same pathway as Mec3. Although exposure to HU reduced the viability of *mcm10Δ150* and *MCM10 mec3Δ* mutants to a similar degree, the growth of *mcm10Δ100 mec3Δ* and *mcm10Δ150 mec3Δ* was about a 100-fold further decreased and truncation of the first 150 residues of Mcm10 had a more severe effect than that of the first 100 amino acids (Figure 7B). Therefore, in contrast to UV irradiation, high concentrations of HU caused synergistic growth suppression in both the *mcm10Δ100 mec3Δ* and *mcm10Δ150 mec3Δ* strains. Because the *mcm10Δ100* mutation led to such a severe phenotype in the absence of *MEC3*, we concluded that the first 100 residues of Mcm10 execute a function that is only revealed in the absence of a functional 9-1-1 clamp. Thus, Mcm10 and Mec3 appear to have overlapping and distinct functions in responding to HU-mediated replication stress.

When we tested CPT sensitivity, a different pattern emerged (Figure 7C). We observed an approximately 10-fold reduction in the ability to resist CTP in the *mcm10Δ150* strain. The elimination of *MEC3* caused strains to be even more sensitive to high levels of CPT than the *mcm10Δ150 MEC3* strain, but in the absence of Mec3, the truncation mutations of Mcm10 had no additional effect. To ascertain that the *mcm10Δ150* strain was indeed less sensitive than the *mec3Δ* mutant, we confirmed these results partially by a more quantitative liquid assay in which cells were dosed with CPT for several hours and then plated onto rich medium to assess colony numbers. Indeed, the *MCM10* and *mcm10Δ100* strains were not sensitive to CPT treatment (Supplementary Figure S8), and although survival of the *mcm10Δ150* strain was modestly compromised, these cells were not as sensitive to the drug as *mec3Δ* mutants. These results suggested that the N-terminus of Mcm10 contributes to resisting CPT-induced damage in the presence of the 9-1-1 clamp. In the clamp's absence, however, the N-terminus of Mcm10 does not play any role.

The last DNA-damaging agent we applied was PLE to induce chromosome breakage. As before, the truncation of the N-terminal 100 amino acids had no effect on survival (Figure 7D). In contrast, the *mcm10Δ150* mutant displayed an approximately 10- to 50-fold higher sensitivity. Notably, this sensitivity was largely independent of *MEC3*, as deletion of this gene had very little effect on the growth behavior of the strains. Therefore, Mcm10 functions in resistance to PLE-induced damage, and this function depends on its N-terminal 150 residues. Since the loss of Mec3 had almost no effect on cell viability, the N-terminus of Mcm10 must have other, Mec3-independent roles in PLE resistance.

## DISCUSSION

### Mcm10 interacts with 9-1-1

In this study, we present data that describe a novel interaction between the replication factor Mcm10 and the 9-1-1 clamp. Two-hybrid experiments suggest that Mcm10 binds specifically to a single subunit of the clamp, Mec3, via its PIP box and residues in its less well-characterized N-terminus. Previous work in our laboratory characterized the function of Mcm10's PIP box in PCNA binding (15). It appears that this motif has also some relevance for binding to Mec3 (Figure 1C). Structural work has established that the PIP box binding pocket, the so-called hydrophobic pocket, is absent in hsRad1 (scRad17), but present in hsRad9 (scDdc1) and—in a spatially more constricted manner—in hsHus1 (scMec3). Theoretically, this makes a PIP box-mediated interaction possible in these two subunits (30), although this is almost certainly weak for homologs of hsHus1 and their respective binding partners. In the light of these considerations, it is not surprising that we identified a second contact site in Mcm10 that is distinct from its PIP motif. Other 9-1-1 interacting proteins, such as FEN1, also have a designated binding site that is non-overlapping with the PIP box (30). Our work identifies a 50-amino acid region within the N-terminus of Mcm10 as a 9-1-1 binding surface that is distinct from Mcm10's self-interaction domain located in the first 100 amino acids of the protein (17,21). It is conceivable that redundant helical turns within the first 150 residues can mediate binding to Mec3 and this may explain why the ID between positions 100 and 150 did not abrogate the Mcm10:Mec3 two-hybrid interaction (Figure 3B). Our data also identify a potential Mcm10 binding surface on Mec3 in its IDL (Figure 4). The corresponding region on PCNA is important for binding to its many substrates (in addition to the nearby hydrophobic pocket formed in its C-terminus). Altogether, these *in vitro* data lead us to propose that the N-terminus of Mcm10 in conjunction with the more centrally located PIP motif binds specifically to the Mec3 subunit of the 9-1-1 clamp. Besides exonuclease 1 (Exo1), Mcm10 is only the second protein that has been identified to bind to Mec3, but not the other two subunits of 9-1-1 (38). Whether Exo1 competes with Mcm10 for the same binding site or interacts with 9-1-1 in response to replication stress, like Mcm10, remains currently unknown. We observed Co-IP of Mcm10 and Mec3 in response to HU and UV light, but not after exposure to CPT, PLE or in untreated cells (Figure 5A). These results suggest that the complex is unstable in the absence of a DNA substrate for 9-1-1, explaining the small amounts of Mec3 and Ddc1 that were pulled down with Mcm10 in DNAse I-treated extracts (Figure 5A and Supplementary Figure S4). Unfortunately, we were unable to perform the Co-IP in the reverse orientation and the question of whether Mcm10 requires modification by ubiquitin, which is necessary for binding to PCNA (15), remains to be answered. Nevertheless, the fact that 9-1-1:Mcm10 complex formation is stimulated by UV irradiation and nucleotide depletion, but not in response to DNA:TopoI adducts or DSBs is also consistent with our genetic data that support completely and partially overlapping roles for Mcm10 and Mec3 in UV light (Figure 7A and Figure 5) and HU resistance (Figure 7B), respectively, and clearly independent roles in resistance to CPT and PLE (Figure 7C and D).

### The N-terminus of Mcm10 plays a role in checkpoint activation in response to nucleotide shortage, but not in response to UV-, CPT- or PLE-induced DNA damage

Our laboratory and others have identified Mcm10 as a strong protector of genome stability, although it is unclear to which exact molecular function of Mcm10 this ability is tied (3–8). The discovery that Mcm10 can bind to the 9-1-1 clamp when stimulated by particular types of genotoxic stress led us to explore the hypothesis that the 9-1-1:Mcm10 interaction promotes checkpoint activation. To our surprise, the *mcm10Δ150* mutant displayed robust checkpoint activation in response to UV light and PLE, but not in the presence of HU (Figure 6C and D). In agreement with other studies, we observed minimal checkpoint activation in response to CPT (36,37). We conclude that the N-terminal 150 amino acid domain of Mcm10 is not required for proficient checkpoint activation, except when replication forks encounter limiting nucleotide pools (Figure 6C and D). In this context, Mcm10 may help to guide the 9-1-1 clamp to its proper substrate, thereby facilitating activation of the checkpoint. This is consistent with the observation that *mcm10Δ150* mutants phenocopy *mec3Δ* cells at high concentrations of HU (Figure 7B).

Two partially overlapping branches of checkpoint activation in S phase have been described in response to HU: one utilizes the 9-1-1 clamp and the other Dpb11 (39,40). The presence of *DPB11* in the *mec3Δ* strains could explain the residual Rad53 phosphorylation observed in response to replication stress or DNA damage (Figure 6C, lower panel). The untreated *mcm10Δ150* strain exhibited a constitutive low-level activation of Rad53 (Figure 6C and D). This was not dependent on *MEC3*, and became more apparent upon release from G1 phase (Figures 6D). It is possible that overlapping sites in Rad53 are targeted for phosphorylation in untreated *mcm10Δ150* and HU-treated wild-type cells. Further experiments will need to address the identity of these sites and the underlying cause of the observed checkpoint activation in the *mcm10Δ150* mutant.

### Mcm10 is required for resistance to genotoxic stress in both a 9-1-1-dependent and -independent manner

**Figure 8.**
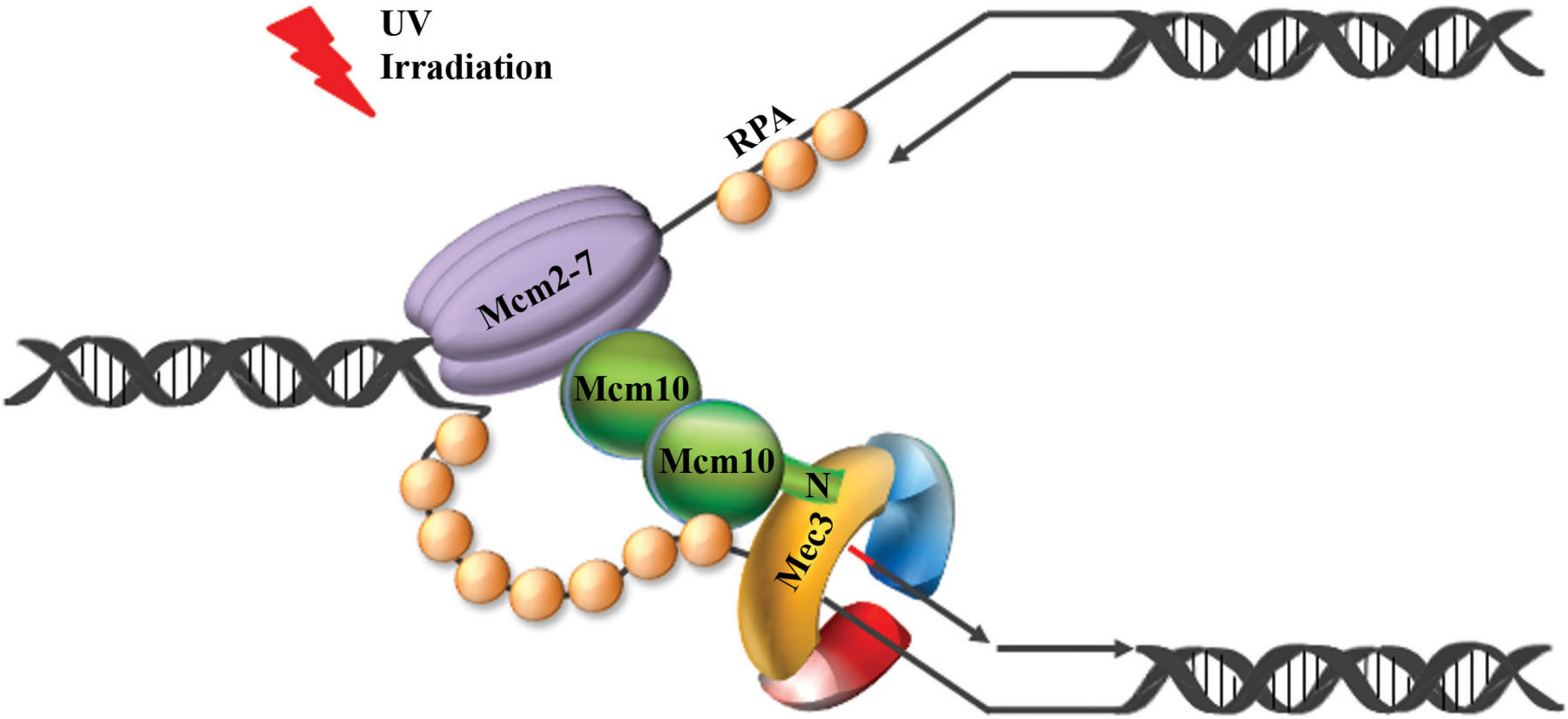
The N-terminus of Mcm10 interacts with Mec3 in response to UV irradiation and may help to tether the 9-1-1 clamp to the replisome to increase fork stability. A model of a stalled replication fork is shown, illustrating the Mcm2-7 core helicase on the leading strand template, RPA coating single-stranded DNA and the 9-1-1 clamp encircling 5' recessed DNA. The N-terminus of Mcm10 (N) interacts with the Mec3 subunit of 9-1-1 (yellow), thereby holding the clamp in place and protecting the encircled Okazaki fragment (depicted with a red RNA primer) from degradation.

We provide strong evidence that the N-terminal region spanning residues 100–150 is important for Mcm10's interaction with the 9-1-1 clamp (Figure 2C and Supplementary Figure S6). Surprisingly, survival of the *mcm10Δ150* strain was affected differently under each stress condition tested (Figure 7A–D). Compared to the *mcm10Δ100* or *MCM10* strains, the *mcm10Δ150* mutant was at least 10-fold more sensitive to UV light, HU, CPT or PLE. Significant phenotypic differences were observed when we combined the truncation mutants with a *mec3Δ* allele to evaluate the dependence of the observed phenotype on the 9-1-1 clamp. Checkpoint activation in response to UV light is dependent on 9-1-1 in budding yeast, at least in the G1 and G2 phases of the cell cycle (27). UV irradiation caused robust checkpoint activation in the *mcm10Δ150* strain, and the increased sensitivity of the mutant was not exacerbated by deletion of *MEC3* (Figure 7A). This implies that Mcm10 and 9-1-1 operate in the same pathway to resist UV light-induced replication stress. Since Rad53 activation in response to UV light was not compromised in *mcm10 Δ150* mutants (Figure 6C), the defect must be due to a checkpoint-unrelated dysfunction that pertains either to the repair or replication of pyrimidine dimers. Indeed, a non-canonical function in error-free postreplicative repair has been proposed for the 9-1-1 complex recently (38). Moreover, there are conflicting data concerning the possible role that 9-1-1 may play in DNA damage tolerance via recruitment and stimulation of translesion polymerases (41,42). The data presented here are compatible with a model by which 9-1-1 and Mcm10 provide structural support of stalled replication forks (Figure 8). Mcm10 is known to bind to the Mcm2-7 core helicase (11) as well as replication protein A (RPA) (12) and may thus help to tether 9-1-1 to the replisome, thereby protecting the unprocessed 5″ terminus of the most proximal Okazaki fragment from resection. We speculate that Mcm10 binding to Mec3 could actively hinder the recruitment of the 5″ to 3″ nuclease Exo1, providing an additional shield to nascent DNA processing at replication forks (43). Although we depict Mcm10 as a dimer in Figure 8, we wish to point out that self-interaction is not required for UV light resistance, as *mcm10*Δ*100* cells did not exhibit any enhanced sensitivity to UV light (Figure 7A). Similarly, Mcm10-mediated protection from DSBs appears to be independent of oligomerization (Figure 7D). However, in this case Mcm10's role is entirely uncoupled from 9-1-1, as a *MEC3* deletion had very little effect on growth in PLE-containing medium and there is no indication of a 9-1-1:Mcm10 interaction under these conditions (Figure 5A). As such, UV light-induced damage is the only form of genotoxic stress we tested that requires the N-terminal tail of Mcm10 solely for interaction with the 9-1-1 clamp. Whether this interaction is conserved in humans is unclear, as hsMcm10 levels are downregulated by ubiquitin-dependent proteolysis in response to high doses of UV irradiation, though in a cell type-specific manner (44,45). Ubiquitin conjugation requires the C-terminus of hsMcm10 (44), which is not conserved in lower eukaryotes (17). Future studies are needed to reveal the relationship between Mcm10 and 9-1-1 in humans.

Unlike the exposure to UV light, treatment with HU resulted in a strong synthetic sickness when either *mcm10Δ100* or *mcm10Δ150* mutants were combined with a *mec3Δ* allele (Figure 7B). Whereas, the *mcm10Δ100* mutant appeared to be checkpoint proficient in HU, *mcm10Δ150* cells were checkpoint compromised (Figure 6C and D) and showed a similar growth defect as *mec3Δ* mutants (Figure 7B). This could be interpreted to mean that checkpoint deficiency was due to the loss of 9-1-1:Mcm10 binding. However, the strong synthetic interaction between *mcm10Δ150* and *mec3Δ* argues against this possibility. In fact, this synthetic relationship is reminiscent of the genetic interaction between a *dpb11-1* mutant and any gene encoding either one of the 9-1-1 clamp subunits or the large subunit of their clamp loader, Rad24 (39). Other replication factors such as Sld2 and Polδ have also been implicated in the Dpb11 branch of checkpoint activation (39,40,46–48). Thus, it is possible that *mcm10Δ150* disrupts the Dpb11 branch of checkpoint activation, which is readily exposed in the absence of *MEC3*. Although the interaction between 9-1-1 and Mcm10 seems to make some contribution to cell survival in HU, what is most surprising, is the requirement for Mcm10 self-interaction that is only revealed in the absence of a functional 9-1-1 clamp (Figure 7B). Whether Mcm10 acts as a dimer, trimer or hexamer is still under debate (17,20,21), and how oligomerization contributes to rendering replication more robust under limiting dNTP supply remains to be elucidated. Lastly, the fact that CPT treatment appears to have a 10-fold effect on survival of the *mcm10Δ150* strain, but is not as deleterious as a full deletion of *MEC3* is also consistent with the idea that *mcm10Δ150* does not fully abrogate *MEC3* function. This observation was confirmed by an independent, more quantitative liquid sensitivity assay (Supplementary Figure S8). Unlike mammalian cells, which induce a robust, replication-dependent checkpoint response to CPT (49,50), budding yeast does not activate the checkpoint unless homologous recombination is dysfunctional (36). Therefore, the 9-1-1 clamp must have a checkpoint-independent repair function in CPT-treated cells.

In conclusion, our study has uncovered an unknown role for Mcm10 in resisting or repairing DNA damage. Mcm10 directly collaborates with the 9-1-1 clamp to defy genotoxic stress following UV irradiation, but seems to work independently of 9-1-1 in the prevention or resolution of DSBs. In this context, it is worthwhile to note that Mcm10 has been identified in a complex with other factors implicated in DSB repair—at least in *Xenopus* egg extracts (51).

## SUPPLEMENTARY DATA

Supplementary Data are available at NAR Online.

SUPPLEMENTARY DATA
