# Supplementary material for: The N-terminus of Mcm10 is important for interaction with the 9-1-1 clamp and in resistance to DNA damage
Source: Nucleic Acids Res. 2014 Jun 27;42(13):8389–404. doi: 10.1093/nar/gku479 (PMC4117747; doi:10.1093/nar/gku479)
Supplement: SUPPLEMENTARY DATA [file supp_42_13_8389__index.html]

The N-terminus of Mcm10 is important for interaction with the 9-1-1 clamp and in resistance to DNA damage — SUPPLEMENTARY DATA 

# The N-terminus of Mcm10 is important for interaction with the 9-1-1 clamp and in resistance to DNA damage

## SUPPLEMENTARY DATA

**Files in this Data Supplement:**

- SUPPLEMENTARY DATA
