# Supplementary material for: The N-terminus of Mcm10 is important for interaction with the 9-1-1 clamp and in resistance to DNA damage
Source: Nucleic Acids Res. 2014 Jun 27;42(13):8389–404. doi: 10.1093/nar/gku479 (PMC4117747; doi:10.1093/nar/gku479)
Supplement: SUPPLEMENTARY DATA [file supp_gku479_nar-00825-d-2013-File010.pdf]

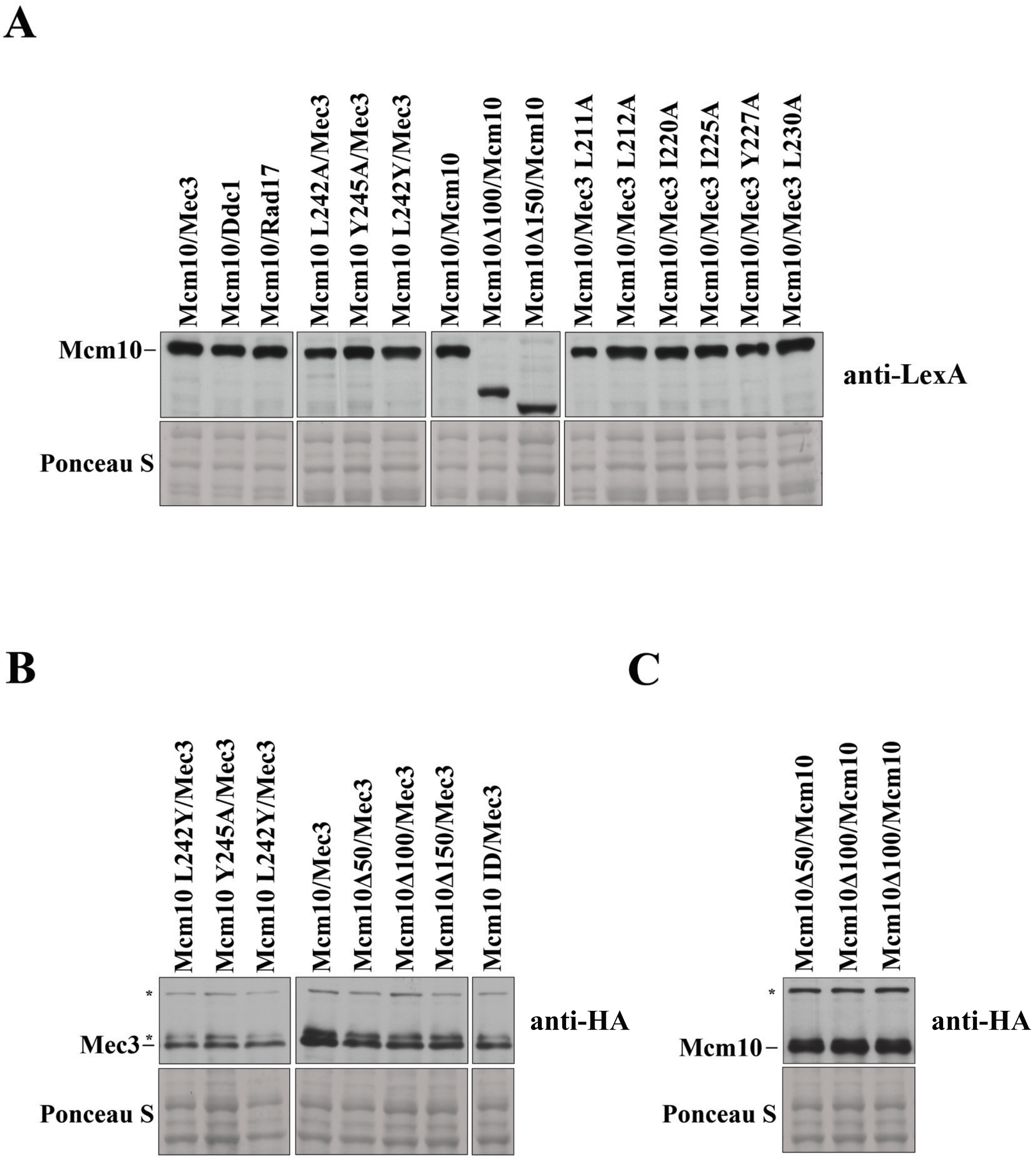

Supplementary Figure S1. Protein expression in yeast two-hybrid strains. (A-C) Immunoblots using an anti-LexA (Abcam, ab14553) or an anti-HA antibody (Covance, 16B12) were used to verify proper expression of the Mcm10 and Mec3 fusion proteins, respectively. The fusion proteins are indicated above each lane as LexA/Gal4-HA pairs. The asterisks indicate bands of uncertain origin. Ponceau S staining served as a loading control.

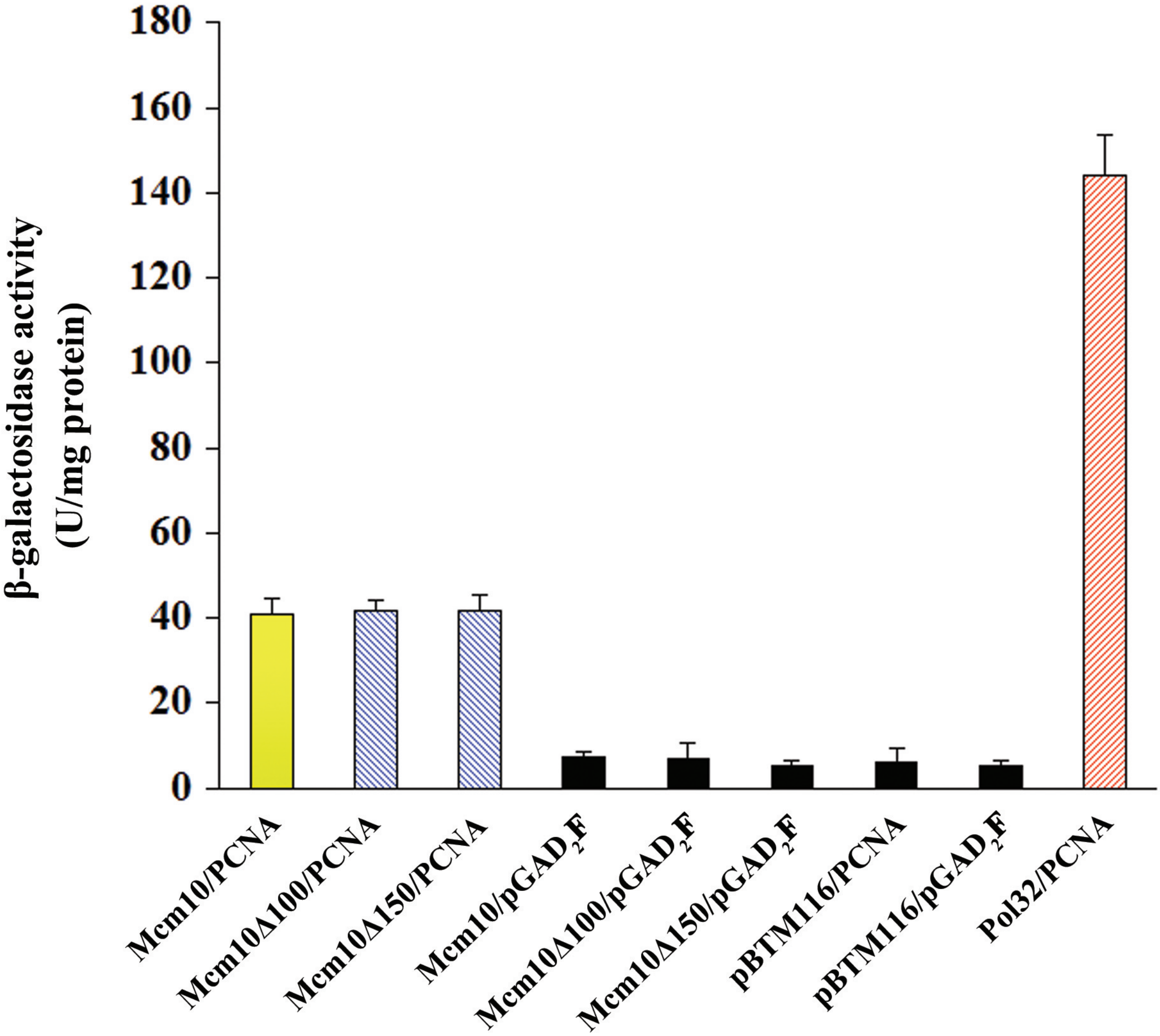

Supplementary Figure S2. Mcm10 truncation mutants interact with PCNA at the same level as wild-type Mcm10. β-galactosidase activity was measured in cell extracts obtained from yeast two-hybrid strains expressing Mcm10, Mcm10Δ100, and Mcm10Δ150 fusion proteins that co-expressed PCNA. Pol32 and PCNA served as a positive control, and extracts expressing the pGAD2F and pBTM116 empty vectors served as negative controls. Each combination was tested in triplicate with three individual transformants. Error bars indicate standard deviations.

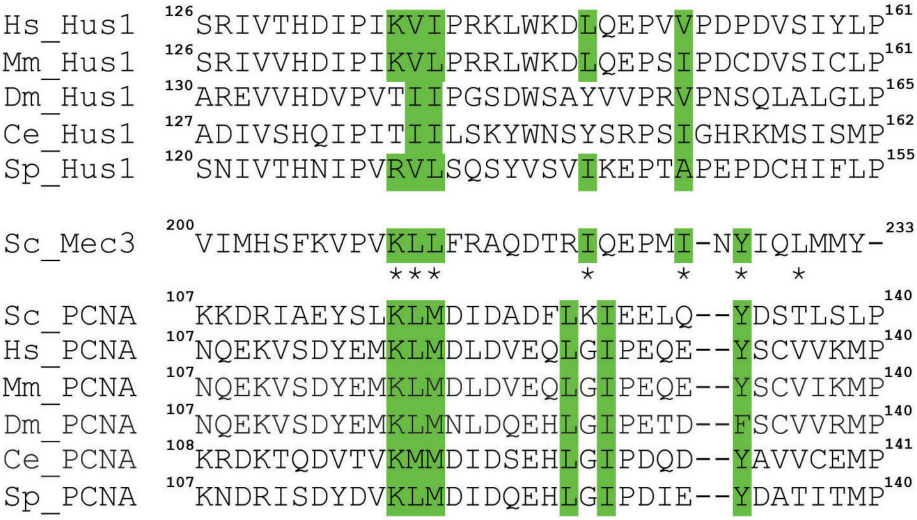

Supplementary Figure S3. A multiple sequence alignment of Mec3/Hus1 and PCNA orthologs from *Homo sapiens* (Hs), *Mus musculus* (Mm), *Drosophila melanogaster* (Dm), *Caenorhabditis elegans* (Ce), *Schizosaccharomyces pombe* (Sp), and *Saccharomyces cerevisiae* (Sc). This figure is a composite from a published alignment (35) with additional PCNA sequences from Mm, Dm, and Sp aligned to HsPCNA using ClustalW (52). Similar residues are highlighted in green, and starred ScMec3 residues indicate they were selected for mutation to alanine.

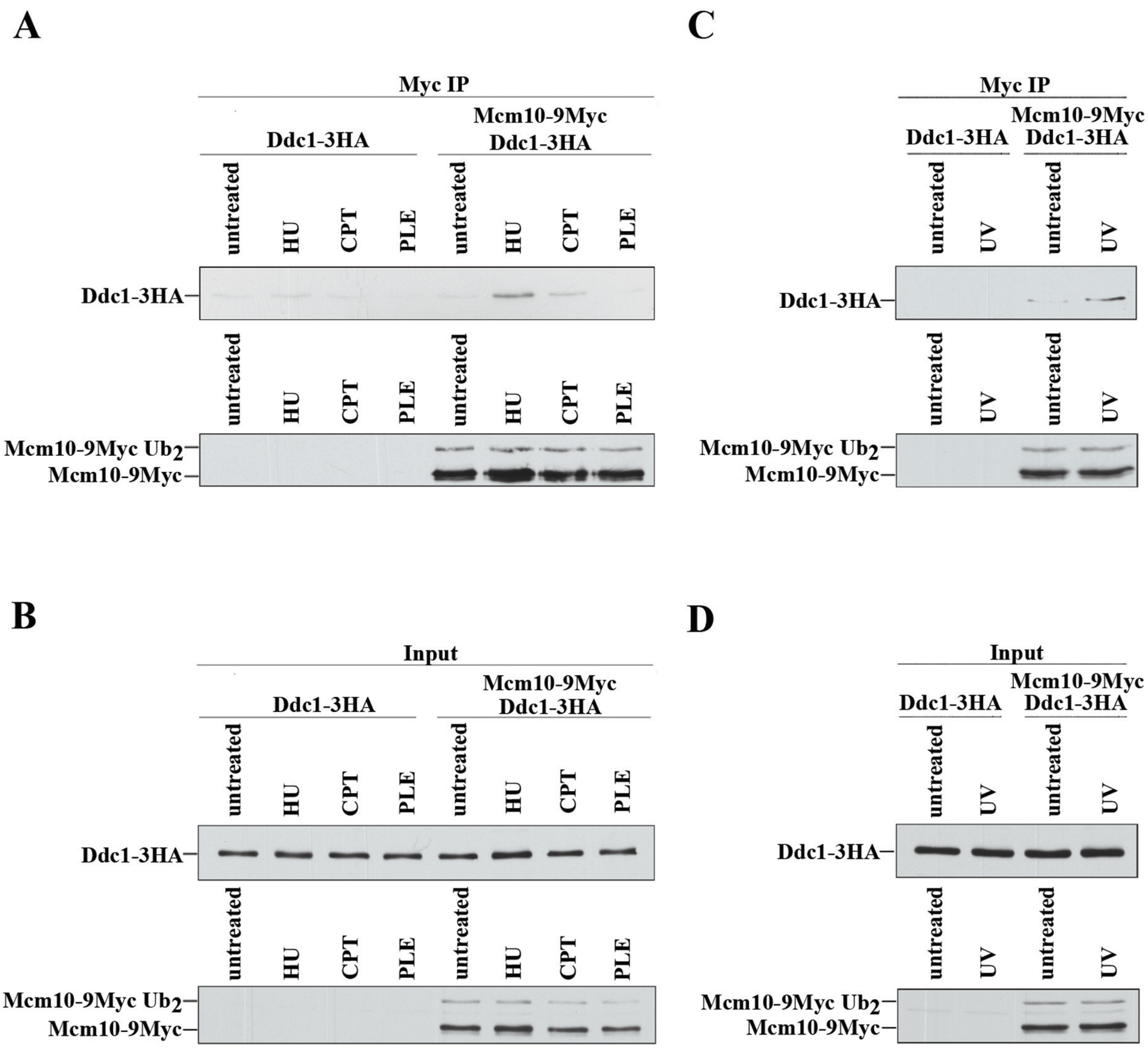

Supplementary Figure S4. Mcm10 interacts with Ddc1 in response to DNA damage and replication stress. AByb1961 (*DDC1-3HA*) cells or AByb925 (*DDC1-3HA, MCM10-9MYC*) cells were grown to log phase and treated with 100 J/m^2 UV, 200 mM HU, 50 mg/mL PLE, or 100 mM CPT as indicated for 1 hr. Cells were harvested, WCEs were prepared, and subjected to immunoprecipitation. Proteins were detected by immunoblot using an anti-HA (Roche, 3F10) or anti-Myc antibody (Thermo Scientific, 9E11). IP lanes are shown in A) and C), input lanes are shown in B) and D). The input lanes represent 1/20 of the IP lanes.

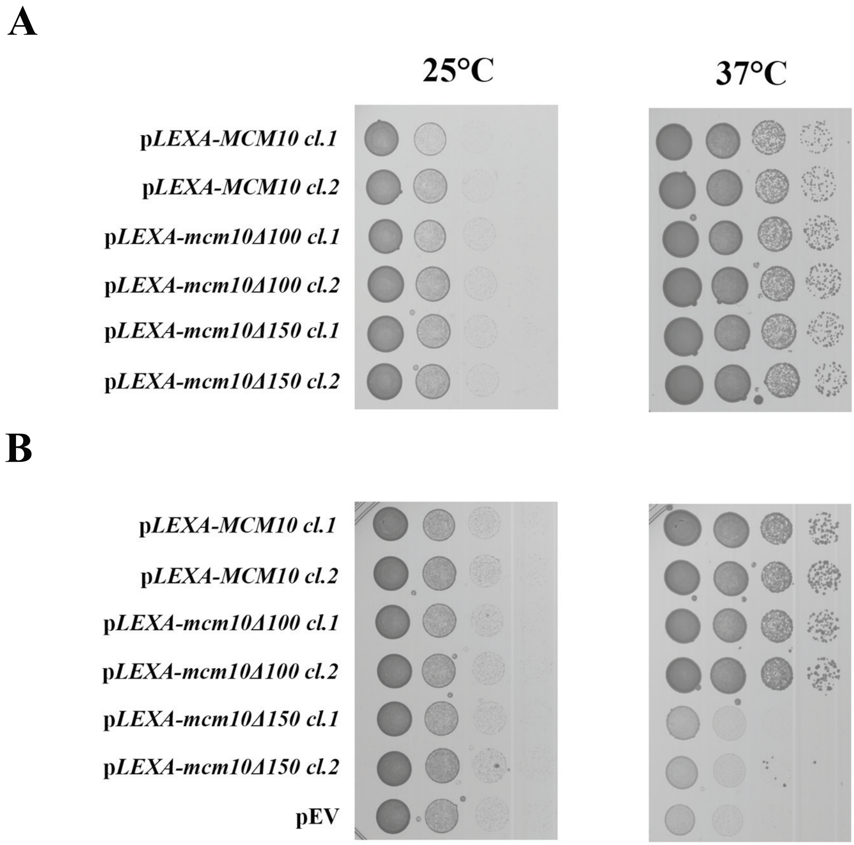

Supplementary Figure S5. Functional complementation of different LexA-tagged *MCM10* constructs. *mcm10-1* cells expressing LexA-tagged full length or truncated Mcm10 were grown to saturation. Serial, 10-fold dilutions of those cells were spotted on plates and incubated at 25 or 37°C for 2 days. In panel A, expression of Mcm10 was controlled by the *ADH1* promoter, which is a constitutively active promoter. In panel B, expression of Mcm10 was controlled by the endogenous *MCM10* promoter.

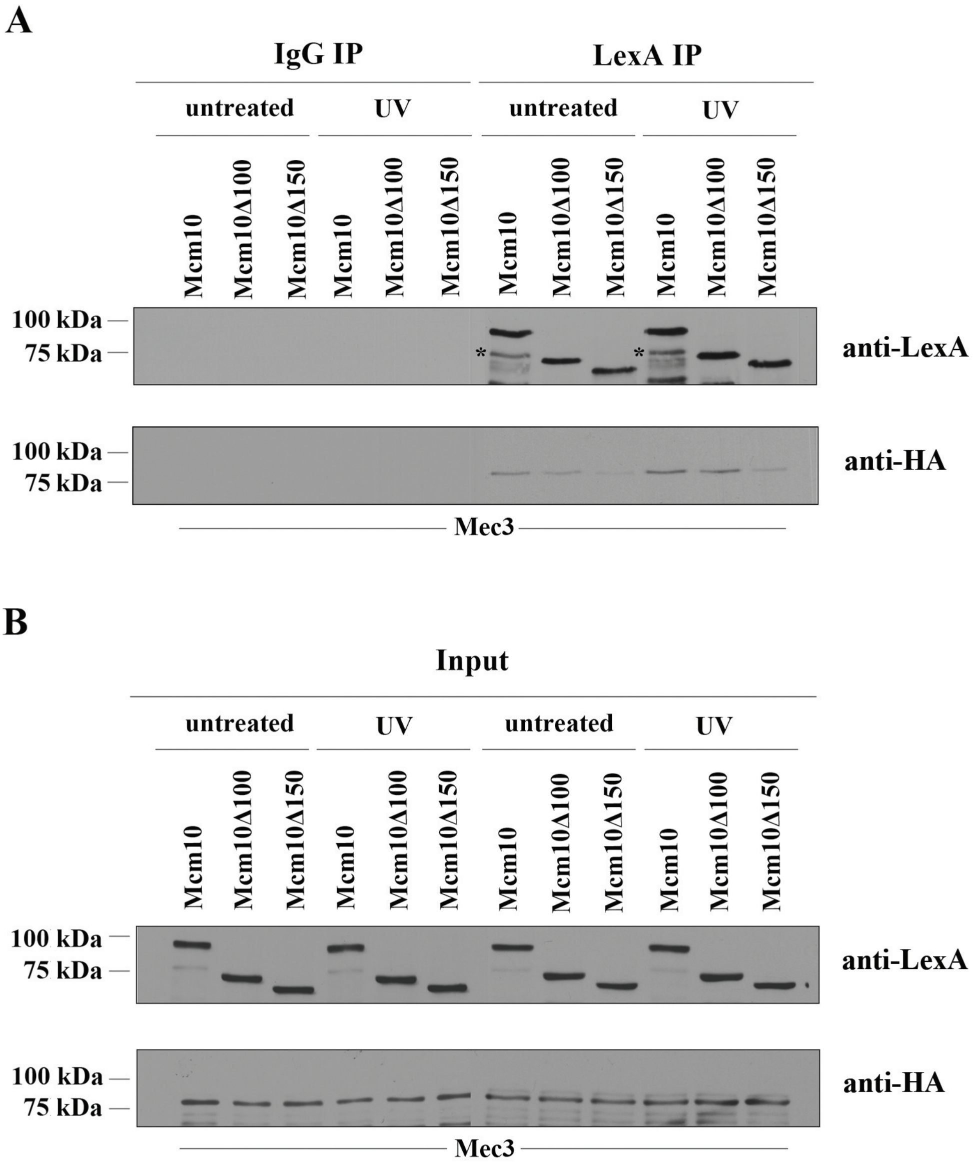

Supplementary Figure S6. Interaction with Mec3 depends on residues 100-150 in the N-terminus of Mcm10. Aby2080 (*LEXA-MCM10, MEC3-HA*), Ab2081 (*LEXA-mcm10 Δ100, MEC3-HA*) and Aby2082 (*LEXA-mcm10Δ150, MEC3-HA*) were grown to log phase and treated with 100J/m^2 UV or left untreated for 30 min. Cells were harvested, WCEs were prepared, and subjected to immunoprecipitation with anti-LexA or IgG antibodies. Proteins were detected by immunoblot using an anti-HA (Roche, 3F10) or anti-LexA antibody (abcam ab14553). IP lanes are shown in A) and input lanes are shown in B). The input lanes are 1/20 of the IP lanes. The asterisks in lanes 7 and 10 indicate bands which are likely degradation products. Please note that in these experiments, we were unable to detect di-ubiquitinated Mcm10.

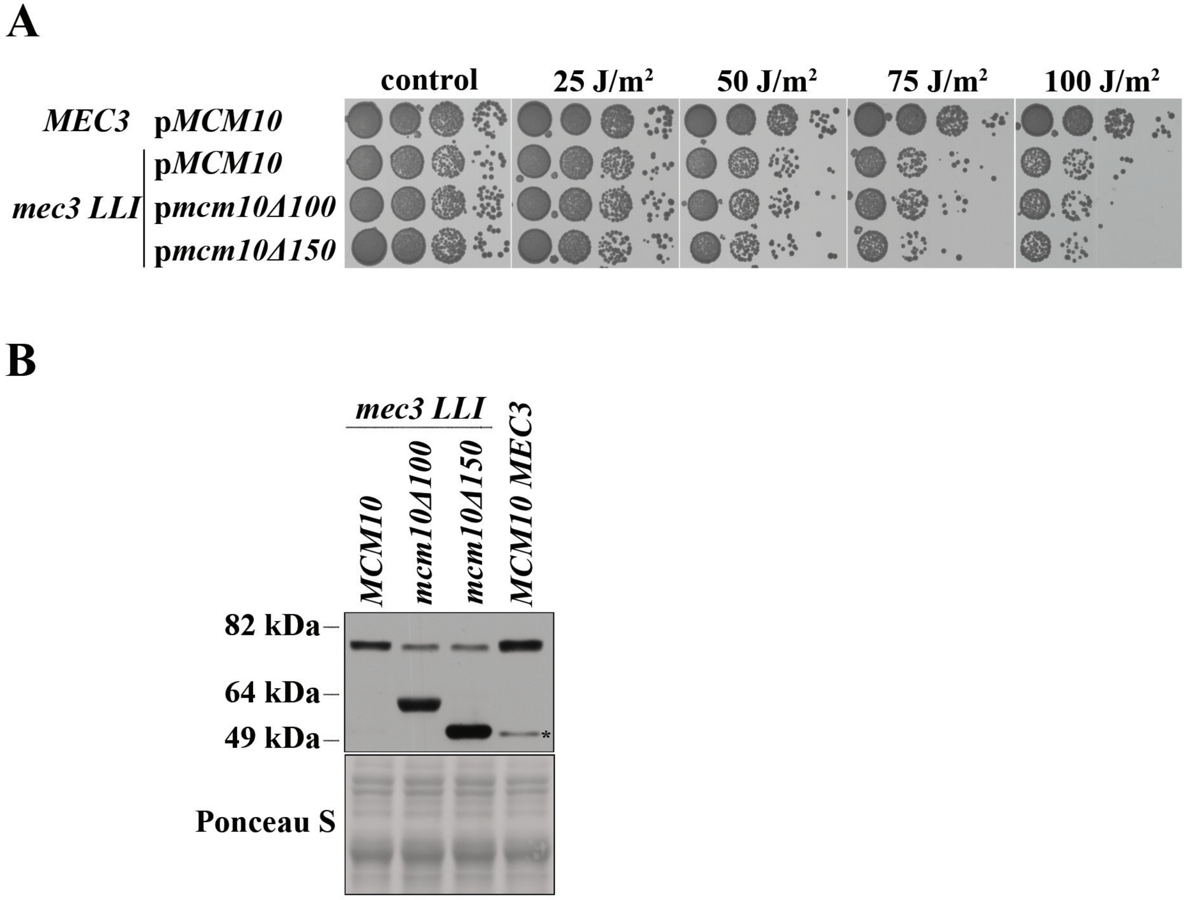

Supplementary Figure S7. *MCM10* and *MEC3* act in a common genetic pathway. A) Serial, 10-fold dilutions of ABy1964 (*MCM10, MEC3*), ABy1967 (*MCM10, mec3 LLI*) ABy1968 (*mcm10Δ100, mec3 LLI*), and ABy1969 (*mcm10Δ150, mec3 LLI*) were spotted on plates, treated with UV light as indicated, and incubated at 30°C for 3 days. (B) Western blot analysis using an anti-HA antibody (Covance, 16B12) was used to verify expression levels of Mcm10, Mcm10Δ100, Mcm10Δ150 as well as Mec3-LLI and Mec3. All proteins were tagged with HA. Wild-type Mcm10 migrates at a similar position as Mec3-LLI and wild-type Mec3 and masks these proteins in lanes 1 and 4. The asterisk in lane 4 indicates a band of uncertain origin, possibly a degradation product of wild-type Mec3, as this was observed in other blots as well (data not shown).

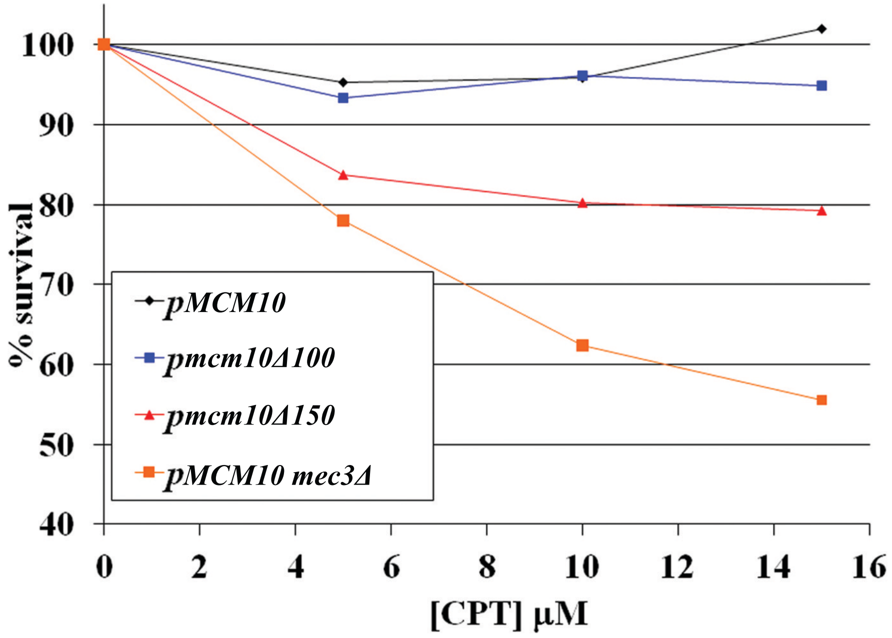

Supplementary Figure S8. The *mcm10Δ150* strain is modestly sensitive to CPT. AByb1487 (*MCM10*), AByb1489 (*mcm10Δ100*), AByb1491 (*mcm10Δ150*), and AByb1576 (*MCM10, mec3Δ*) were grown, treated with indicated amounts of CPT, and plated as described in the Materials and Methods. Percent survival was calculated in reference to the number of surviving colonies in untreated cell cultures.

References

35. Venclovas, C. and Thelen, M.P. (2000) Structure-based predictions of Rad1, Rad9, Hus1 and Rad17 participation in sliding clamp and clamp-loading complexes. *Nucleic Acids Res*, **28**, 2481-2493.

52. Larkin, M.A., Blackshields, G., Brown, N.P., Chenna, R., McGettigan, P.A., McWilliam, H., Valentin, F., Wallace, I.M., Wilm, A., Lopez, R. *et al*. (2007) Clustal W and Clustal X version 2.0. *Bioinformatics*, **23**, 2947-2948.
